# Welsh, González, and Negulescu share Lasker Award recognizing transformative treatments for people with cystic fibrosis

**DOI:** 10.1172/JCI198727

**Published:** 2025-09-11

**Authors:** Benjamin D. Singer, G.R. Scott Budinger

**Affiliations:** 1 Division of Pulmonary and Critical Care Medicine, and; 2 Simpson Querrey Lung Institute for Translational Science (SQ LIFTS), Northwestern University Feinberg School of Medicine, Chicago, Illinois, USA.

The Lasker Foundation has awarded the 2025 Lasker~DeBakey Clinical Medical Research Award to Michael J. Welsh, Jesús (Tito) González, and Paul Negulescu for the development of a novel class of drugs that nearly completely reverses the signs and symptoms of cystic fibrosis (CF) and slows disease progression. This award recognizes a translational tour-de-force, bridging the fundamental discovery of the molecular basis of CF in the 1980s to well-tolerated oral therapies that have revolutionized the lives of many thousands of people with CF in the 21st century.

## The clinical manifestations and molecular pathogenesis of cystic fibrosis

In 1938, a pathologist, Dorothy Andersen, coined the term “cystic fibrosis of the pancreas” after identifying fluid-filled pancreatic cysts surrounded by scarring in autopsy specimens from children with a presumed diagnosis of celiac disease (1). Cystic fibrosis (CF) is now recognized as a multisystem disease — including profound effects on the exocrine and endocrine pancreas, liver, reproductive organs, and other organ systems — but most of the morbidity and mortality associated with CF arises from its effect on the conducting airways of the lungs. Patients with CF develop severe, progressive bronchiectasis, mucus hypersecretion, and chronic bacterial infection of the airways that ultimately cause respiratory failure (2). Advances in supportive care, including nutritional interventions, devices and drugs to support airway clearance, protocol-driven use of antibiotics, and lung transplantation, have allowed patients with CF to survive beyond their teenage years. Nevertheless, therapies targeting the fundamental pathobiology of CF remained elusive until the work of this year’s Lasker~DeBakey awardees brought fundamental discoveries in the laboratory to the clinic.

A seminal report from Paul Quinton in 1983 identified that the sweat ducts of people with CF had low permeability to chloride ions, leading to impaired salt reabsorption and high sweat chloride levels (3), the latter representing the foundation of clinical testing for CF diagnosis since 1959 (4). In the same year, in work published by the *JCI*, Michael Knowles identified similar abnormalities in chloride transport in the airways of patients with CF (5). Two years later, Welsh and colleagues at the University of Iowa digested tracheal mucosa from a 12-year-old girl who had died from CF and used the epithelial cells to localize the abnormality in chloride permeability to the airway epithelial cell apical membrane (6). A watershed advancement in understanding the molecular basis for these observations came in 1989, when Francis Collins and Lap-Chee Tsui led a group in Toronto in cloning and characterizing the gene encoding the cystic fibrosis transmembrane conductance regulator (*CFTR*) on chromosome 7 (7). Soon after, Welsh and colleagues observed that expression of wild-type CFTR corrected defective chloride transport in CF airway epithelial cells, whereas expression of CFTR harboring the most common mutation in the United States, F508del, which fails to traffic to the apical cell surface, did not correct the abnormality (8). Within a year of that discovery, Welsh and colleagues reported that CFTR is a cAMP-regulated anion channel for chloride (9), firmly establishing the molecular pathology underlying CF disease. A breakthrough observation from Welsh’s laboratory — that reduced temperature rescues trafficking of mutant CFTR-F508del to the cell surface — provided proof of concept that mutant CFTR could undergo functional rescue (10). These discoveries leveraged the cutting-edge molecular biology techniques of the day to rapidly identify the genetic and biochemical basis of CF disease, tying defective chloride transport to impaired cellular function and providing a footing to understand the ways in which mutant CFTR might be therapeutically targeted.

## Spectrum and classification of CFTR mutations

Systematic genetic sequencing of people with CF revealed the complexity and heterogeneity of CF mutations that cause autosomal recessive disease. While *F508del* accounts for approximately 85% of *CFTR* mutations in the United States, more than 700 disease-causing mutations (>2,000 total) have been identified and grouped into 6 classes (11). Class I mutations result in premature stop codons with reduced or absent CFTR protein expression. Class II mutations, including *F508del*, result in protein misfolding and premature degradation before CFTR can traffic to the apical cell surface. Class III mutations, including *G551D*, reduce the open time of the chloride channel (i.e., impaired gating). Class IV, V, and VI mutations result in impaired channel conduction, insufficient protein production, and reduced cell-surface stability, respectively. Despite its limitations, including that some mutations, such as F508del, exhibit features of multiple classes, this mutation classification framework offers insights into mechanistic targets for the therapies pioneered by this year’s Lasker~DeBakey awardees.

## The porcine model of CF

While mice harboring mutated *CFTR* offered mechanistic insights into the pathobiology of CF, they do not develop the disease phenotype in airways and other tissues that is typically found in people with CF. To address this limitation, Welsh and colleagues used somatic cell nuclear transfer to generate a porcine model of CF, which recapitulated nearly all the clinical features of CF disease observed in human newborns (12). Critically, CF pigs bearing CFTR-F508del go on to develop intestinal, pancreatic, and airways pathology resembling that observed in people with CF (13). An important early insight from Welsh’s porcine model was that airway epithelia in newborn CF pigs do not hyperabsorb sodium (14), contrasting with a prevalent hypothesis at the time that CFTR inhibits the epithelial sodium channel to dehydrate the airways (15). Other animal models of CF, including ferrets (16), have provided additional insights into CF disease pathogenesis that will help to identify therapeutic targets.

## Development of CFTR modulators

Leveraging the Welsh discoveries, biotechnology companies in the mid-late 1990s launched drug discovery programs aiming to modulate CFTR. In 2000, the Cystic Fibrosis Foundation invested $30 million in Aurora Biosciences for the development of new drugs to treat CF. Vertex Pharmaceuticals acquired Aurora Biosciences in 2001.

Jesús González and Paul Negulescu worked for Aurora Biosciences and joined Vertex Pharmaceuticals during the 2001 acquisition. Work by González and Negulescu at Vertex applied results from screening compound libraries in CF primary airway epithelial cultures to rescue CFTR-F508del trafficking and gating (17). These discoveries led Negulescu and colleagues to develop VX-770 (later named ivacaftor), which rescues CF airway epithelial cell function in vitro by potentiating the activity of mutant CFTR-G551D (18). His group at Vertex went on to develop VX-809 (lumacaftor), which promotes proper CFTR folding and membrane trafficking of CFTR-F508del (19).

## The clinical revolution: CFTR potentiators and correctors

Ivacaftor works as a potentiator to augment CFTR channel gating and is effective in people with CF whose mutations cause a reduction in channel opening. Accordingly, ivacaftor was initially shown in randomized clinical trials to be efficacious in patients with the class III mutation *G551D*, resulting in dramatic improvements in lung function, exacerbation risk, and nutritional status (20, 21). Unfortunately, because of the high prevalence of the *F508del* (class II/trafficking) mutation, only about 1 in 20 people with CF carry mutations that are correctable with ivacaftor monotherapy.

**Figure 1:**
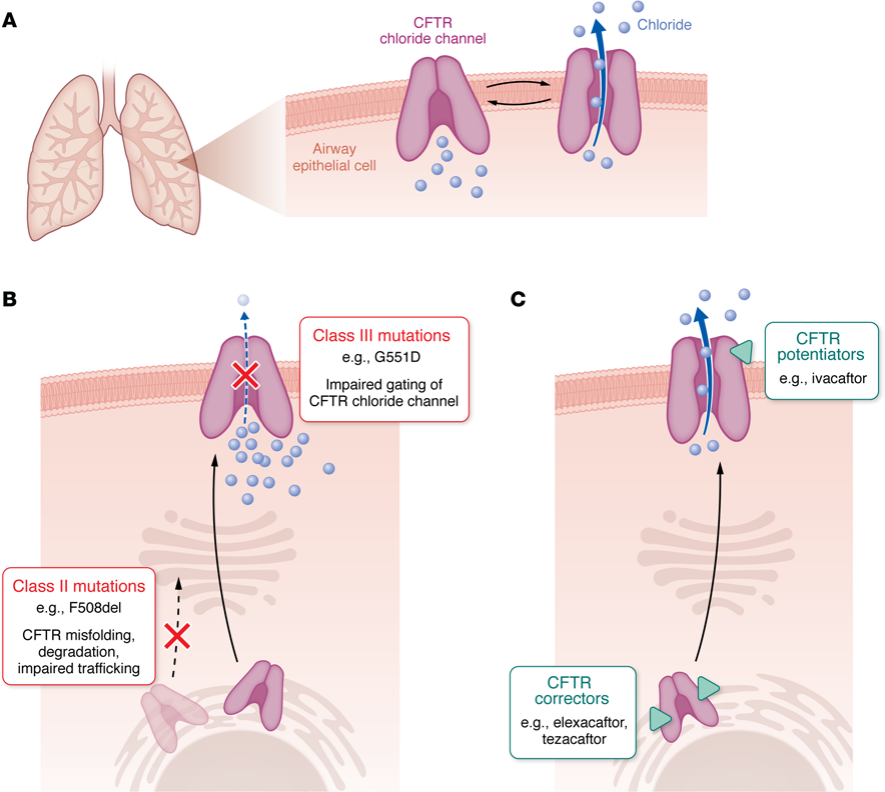
Mechanisms of action of CFTR modulators. (**A**) Normal CFTR function permits conductance of chloride anions. (**B**) Class II mutations (e.g., F508del) cause misfolded CFTR and impair trafficking to the apical cell membrane. Class III mutations (e.g., G551D) impair opening (gating) of the CFTR chloride channel. (**C**) CFTR correctors (e.g., elexacaftor, tezacaftor) promote proper protein folding and trafficking. CFTR potentiators (e.g., ivacaftor) enhance channel opening to promote proper channel gating.

Patients with class II mutations, including *F508del*, require combination therapy with corrector drugs that facilitate proper protein folding and trafficking of CFTR to the cell surface, where the potentiator ivacaftor can enhance channel conductance (22–24). The greatest benefits to date have been seen in triple therapy, combining two correctors that promote proper trafficking — elexacaftor and tezacaftor — with ivacaftor to enhance channel conductance of the trafficked protein (Figure 1) (25–27). Triple therapy, also called highly effective modulator therapy, treats CF disease of the lungs and promotes nutritional status, fertility, and function of the pancreas and liver, revolutionizing the lives of many thousands of individuals with CF.

## Concluding remarks

The development of targeted therapeutics for CF, recognized by this year’s Lasker~DeBakey Award, represents the culmination of a decades-long partnership between academia, the NIH, patient advocates, and industry that has driven biomedical research in the United States. These awardees have shown how a relentless focus on human health can bring fundamental discoveries to the clinic that have transformed the lives of people with CF. The radically positive effect of highly effective modulator therapy on the lives of people with CF cannot be understated. Nevertheless, therapies for people with CF who harbor mutations (e.g., class I mutations) that are not amenable to available therapeutics will require innovative pharmacological strategies, including read-through drugs, splice modulators, and other nucleic acid–based therapies, some of which offer promise for a cure. We remain hopeful that the next generation of investigators will be able to use the path forged by Welsh, González, and Negulescu to realize this hope for a cure not only for people with CF but also those with other diseases for which a cure seems a distant hope.

## Version 1 09/11/2025

Electronic publication
